# Comparative Evaluation of the Clinical Efficacy of Four Different Gingival Retraction Systems: An In Vivo Study

**DOI:** 10.7759/cureus.23923

**Published:** 2022-04-07

**Authors:** Rahul Madaan, Jyoti Paliwal, Vineet Sharma, Kamal K Meena, Ashish Dadarwal, Roshni Kumar

**Affiliations:** 1 Department of Prosthodontics, Rajasthan University of Health Sciences College of Dental Sciences, Jaipur, IND

**Keywords:** retraction cord, gingival displacement, retraction system, cordless, gingival retraction

## Abstract

Introduction: There are numerous gingival retraction systems available on the market. This study aimed to evaluate the clinical efficacy of four gingival retraction systems, namely, impregnated retraction cord, gingival retraction capsule, retraction paste, and polyvinyl acetate strips.

Methods: A total of 20 people were chosen for the study, and 100 specimens were collected. The specimens were classified into five groups based on the materials used for gingival displacement. On the first day, a baseline impression without gingival displacement was made. Afterward, impressions were made with any of the following four gingival retraction systems: impregnated retraction cord (SURE-Cord® Plus; Sure Dent Corporation, Jungwon-gu, South Korea), retraction capsule (3M ESPE astringent retraction paste capsule; 3M Corporation, St. Paul, MN), retraction paste (Traxodent® Hemodent® Paste Retraction System; Premier Dental Co., Plymouth Meeting, PA) and polyvinylacetate strips (Merocel; Merocel Co., Mystic, CT), with a 14-day interval between each system. The amount of gingival displacement was measured using an optical microscope as the distance from the tooth to the gingiva crest in a horizontal plane.

Results: All experimental groups had higher gingival displacement than the control group (P < 0.01). Among the experimental groups, polyvinyl acetate strips had the highest gingival displacement value (541.65 μm), followed by impregnated retraction cord (505.37 μm), retraction capsule (333.57 μm), and retraction paste (230.63 μm).

Conclusion: Within the limits of this in vivo study, significant differences in horizontal gingival displacement were discovered among the four evaluated systems. The horizontal displacement requirements of 200 μm were exceeded by all four systems. The maximum value for gingival displacement was found in polyvinyl acetate strips (Merocel), followed by impregnated retraction cord (SURE-Cord), and retraction capsule (3M ESPE), and the lowest value was found in retraction paste (Traxodent).

## Introduction

The primary goal of any prosthodontic restoration is to preserve what remains rather than to replace what is missing [1]. Optimal gingival health is required before, during, and after treatment to achieve this goal. However, for esthetic or functional reasons, restoration margins are frequently located within the gingival sulcus [2]. Subgingival margins tend to increase the potential for periodontal problems (gingival inflammation) [3,4]. Nevertheless, even if there are subgingival margins, periodontal health can be preserved with meticulous soft tissue management and well-fitting, well-contoured crowns [5,6]. The location of the prepared cervical margin within the sulcus is crucial for long-term gingival health and impression making. The margin should be 0.5 mm from the healthy free gingival margin or 3.0-4.0 mm from the alveolar bone crest, and it should follow the natural scalloped contour of the attachment and alveolar housing [7,8].

For subgingival margins, the finish margin should be accurately recorded in an impression [9]. A sulcular width of at least 0.2 mm is essential to minimize tearing of the impression material and a reduction in marginal accuracy [10]. Furthermore, vertical gingival displacement is essential to allow the impression material to record the prepared tooth apical to the finish line. Also, whenever a hydrophobic material is utilized, it is essential to control moisture in the sulcus as it can cause an impression to be defective [11].

There are two types of gingival tissue displacement techniques: non-surgical and surgical. Non-surgical procedures include mechanical and chemicomechanical approaches, whereas surgical methods include lasers, electrosurgery, and rotary curettage. Different clinical settings require the use of a variety of techniques [12]. The most commonly used method is chemomechanical retraction with a retraction cord and a hemostatic agent. Chemicals used in conjunction with retraction cords (gingival displacement medicaments) are broadly classified as vasoconstrictors (epinephrine, sympathomimetic amines) and astringents (aluminium sulphate compounds, aluminum potassium sulphate [alum] and aluminium sulfate, aluminium chloride, ferric sulfate) [13].

Retraction cords, however, are time-consuming and might cause gingival laceration if not utilized properly [14]. This has led to a rise in the use of cordless retraction materials. Expasyl paste (Kerr Corp, Orange, CA), Magic Foam cord (Coltene/Whaledent Inc., Cuyahoga Falls, OH), Racegel (Septodont, Saint-Maur-des-Fossés, France), GingiTrac (Centrix Inc., Shelton, CT), retraction capsule (3M ESPE astringent retraction paste capsule; 3M Corporation, St. Paul, MN), retraction paste (Traxodent® Hemodent® Paste Retraction System; Premier Dental Co., Plymouth Meeting, PA), and other cordless retraction materials are available on the market. The retraction capsule (3M ESPE) contains 15% aluminium chloride in the form of a paste-like material and is provided with a specific dispenser, whereas the retraction paste (Traxodent) contains 15% aluminium chloride in the form of a syringe and is supplied with comprecaps [15].

A polyvinyl acetate strip is a porous synthetic material made from a biocompatible polymer (hydroxylated polyvinyl acetate) with a net-like structure. With the absorption of intracrevicular fluids, this sponge-like material expands, placing moderate pressure on surrounding gingival tissues and ensuring gingival displacement [16,17]. The dentist's choice of optimal gingival retraction system remains a dilemma [16,18]. There have been a few studies that compare the efficacy of gingival retraction based on the materials and techniques used. This in vivo study used impregnated gingival retraction cord (SURE-Cord® Plus; Sure Dent Corporation, Jungwon-gu, South Korea), gingival retraction capsule (3M ESPE), gingival retraction paste (Traxodent), and polyvinyl acetate strips (Merocel; Merocel Co., Mystic, CT) to compare and assess the degree of lateral gingival displacement on an unprepared tooth.

## Materials and methods

This controlled clinical trial enlisted the participation of 20 undergraduate dental students of both sexes. The study was carried out in the Department of Prosthodontics, Rajasthan University of Health Sciences (RUHS) College of Dental Sciences, Jaipur, and the measurements were made at the Materials Research Centre, Malaviya National Institute of Technology, Jaipur. Participants were selected according to the following inclusion criteria: participants between 18 and 30 years of age with a complete set of natural teeth (the third molar may or may not be present), no malocclusion, with no crowding, rotation, or diastema in teeth in the anterior region. Participants with uncontrolled systemic diseases and gingival or periodontal diseases were excluded from the study. Before the study, the approval of the Institutional Ethical Committee (RUHS‑CDS/EC/2019/Proposal/001) of the RUHS College of Dental Sciences, Jaipur, and informed consent of each participant were obtained. The participant data was formulated and used for research purposes. All procedures were thoroughly explained before participation. Unprepared teeth were chosen because mechanical trauma during tooth preparation might produce gingival irritation and inflammation, making comparisons of efficacy unrealistic.

Step I: Making preliminary impressions

**Figure 1:**
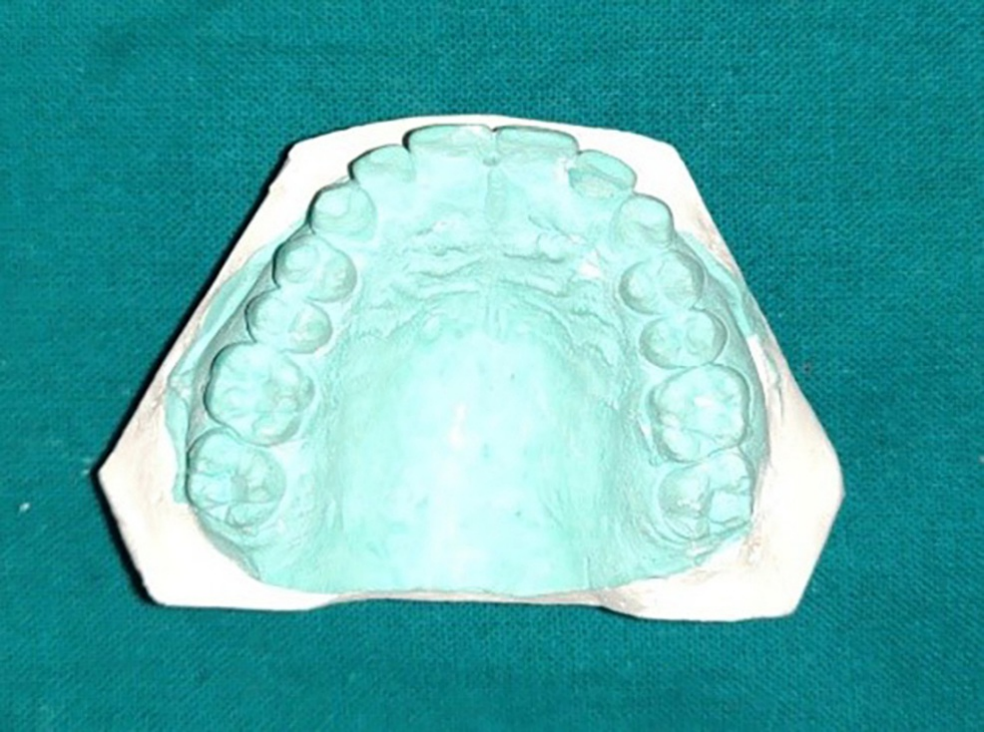
Primary cast

Primary impressions were made on a stock metal tray with irreversible hydrocolloid impression material (Zelgan; Dentsply, New Delhi, India) and were poured in type III dental stone (Kalstone; Kalabhai Karson Private Limited, Mumbai, India) to obtain the primary cast (Figure 1).

**Figure 2:**
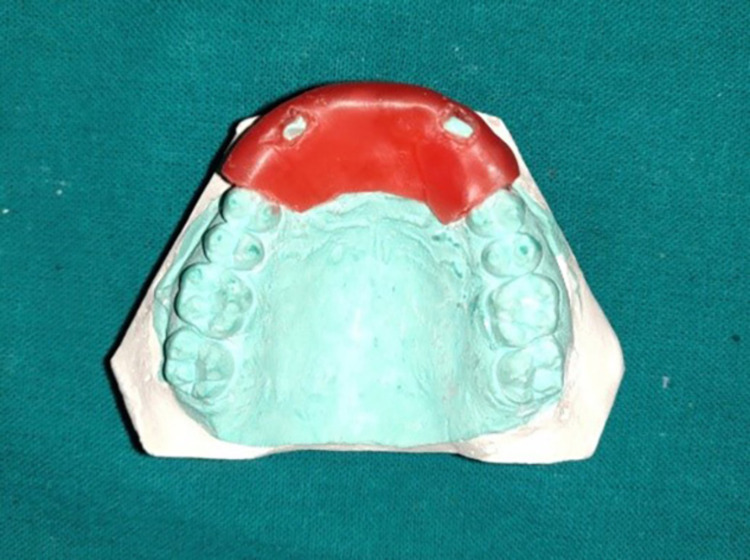
Primary cast with the spacer

**Figure 3:**
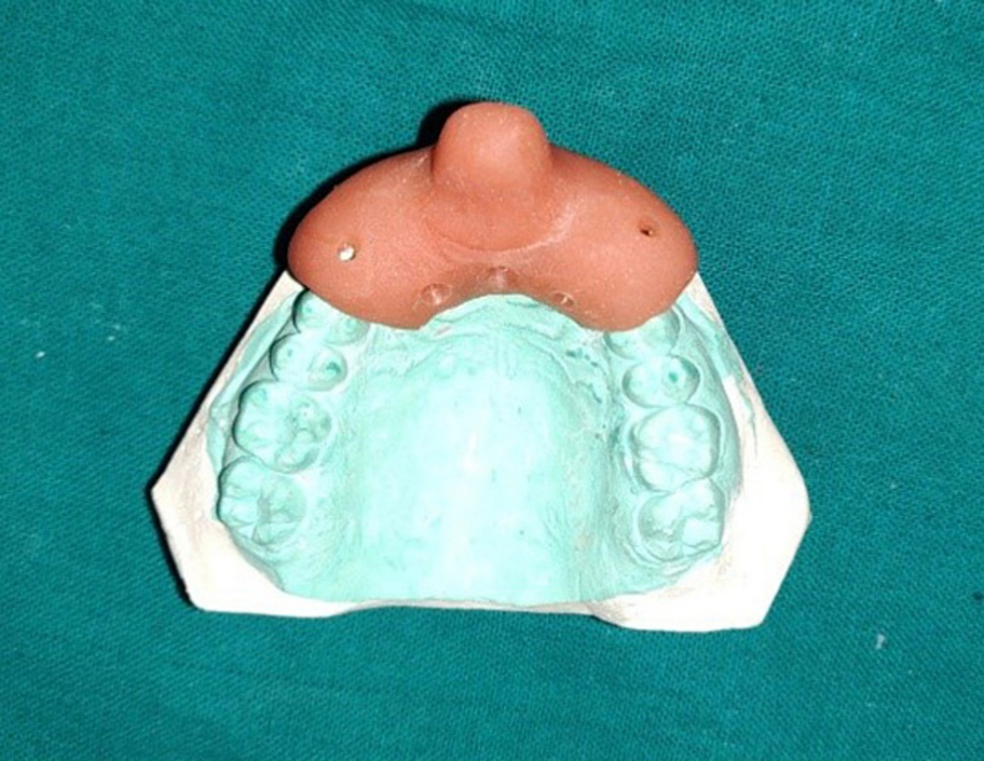
Primary cast with the custom tray

On the maxillary anterior region of the primary cast, a double-thickness spacer (Modeling wax no. 2; MDM Corp, New Delhi, India) was adapted 2 mm short of the vestibular sulcus (Figure 2). On the incisal edges of the maxillary lateral incisors, tissue stops were made to prevent the tray from moving (Figure 2). The custom tray of thickness 2 mm was made by the dough method using an auto-polymerizing resin tray material (Coltocure C; Coltene, Mumbai, India) (Figure 3). After checking the proper extension of the tray in the patient's mouth, the wax spacer was removed and necessary corrections were made.

The custom tray improves the accuracy of elastomeric impression by minimizing the shrinkage caused by the uneven thickness of the material (Figure 3) [19]. In the custom tray, perforations were added to prevent the impression material from separating from the tray. One custom tray was used to make the impression of pre-displaced and post-displaced gingiva with four different gingival retraction systems.

Step II: Pre-gingival displacement impression

**Figure 4:**
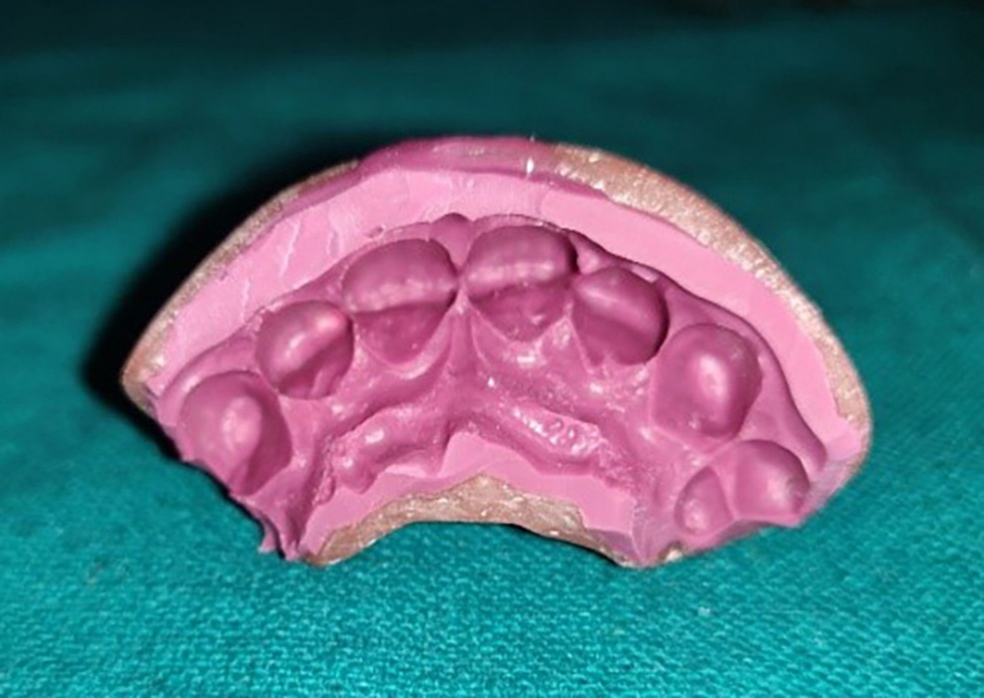
Pre-gingival displacement impression

Pre-gingival displacement impressions were made with the monophase addition silicone impression material (Aquasil Ultra Monophase; Dentsply) using the single-mix-one-step impression technique (Figure 4) [20,21]. An auto-mixing dispensing gun was used to ensure homogeneous mixing between bases and catalysts and to reduce air inclusion during mixing. Initially, the base and catalyst were bled before applying the auto-mixing tip to ensure a free and even flow. To ensure a saliva-free surface, cotton rolls were used to isolate the maxillary anterior area. The intraoral tip was then used to inject addition silicone into the gingival crevice. The material was gently seated in the mouth after being placed in the custom tray.

**Figure 5:**
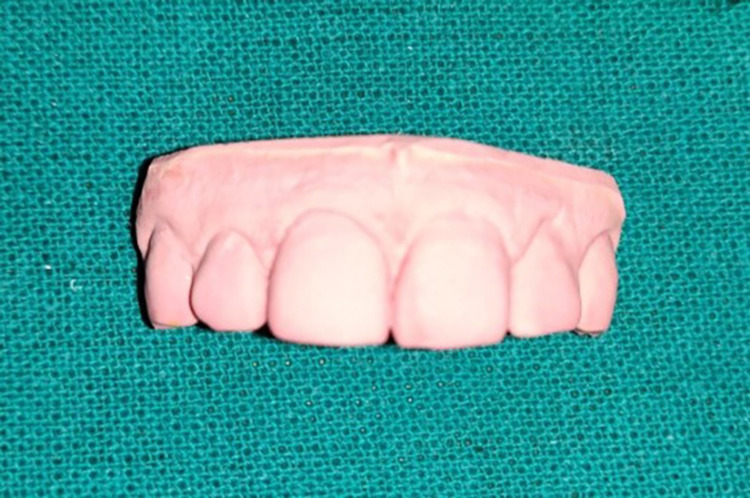
Pre-gingival displacement cast

Impressions were allowed to remain in place without being moved until they set. The trays were removed after five minutes, as specified by the manufacturer. The impressions were analyzed under magnification for voids and streak-free impressions with adequate extensions. Following removal, the impressions were rinsed under running tap water and disinfected in a 5.25% sodium hypochlorite solution for 10 minutes. The impression was then poured with type IV dental stone (Kalrock, Kalabhai Karson Private Limited) (Figure 5).

Step III: Intraoral preparation for gingival retraction

**Figure 6:**
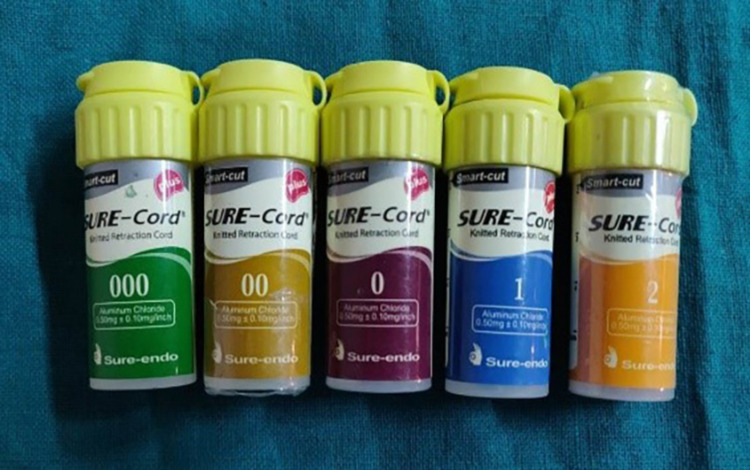
Retraction cord (SURE-Cord)

**Figure 7:**
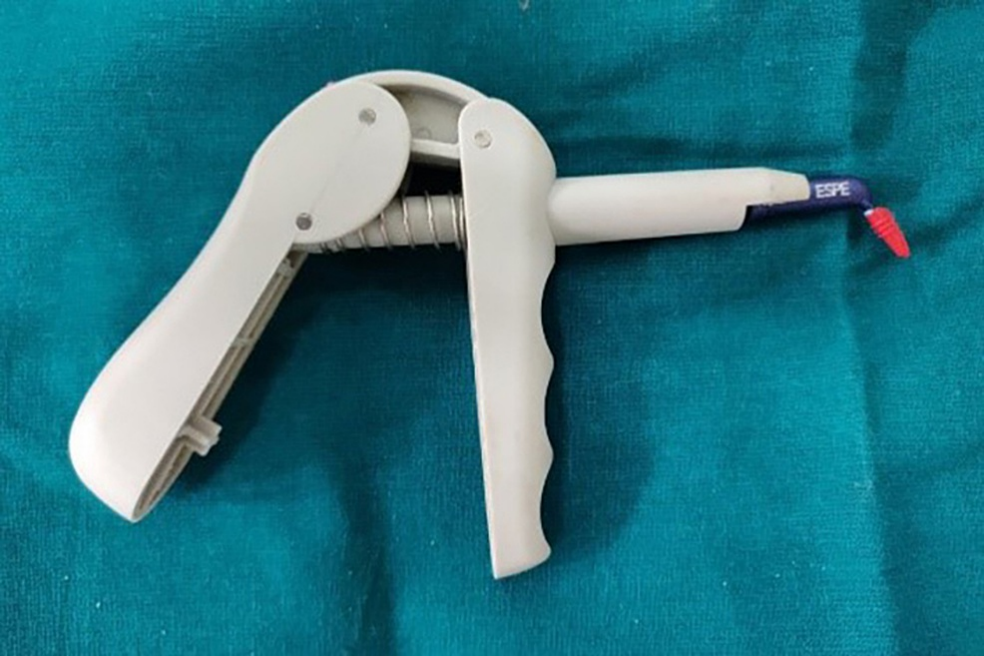
3M capsule with the dispenser

**Figure 8:**
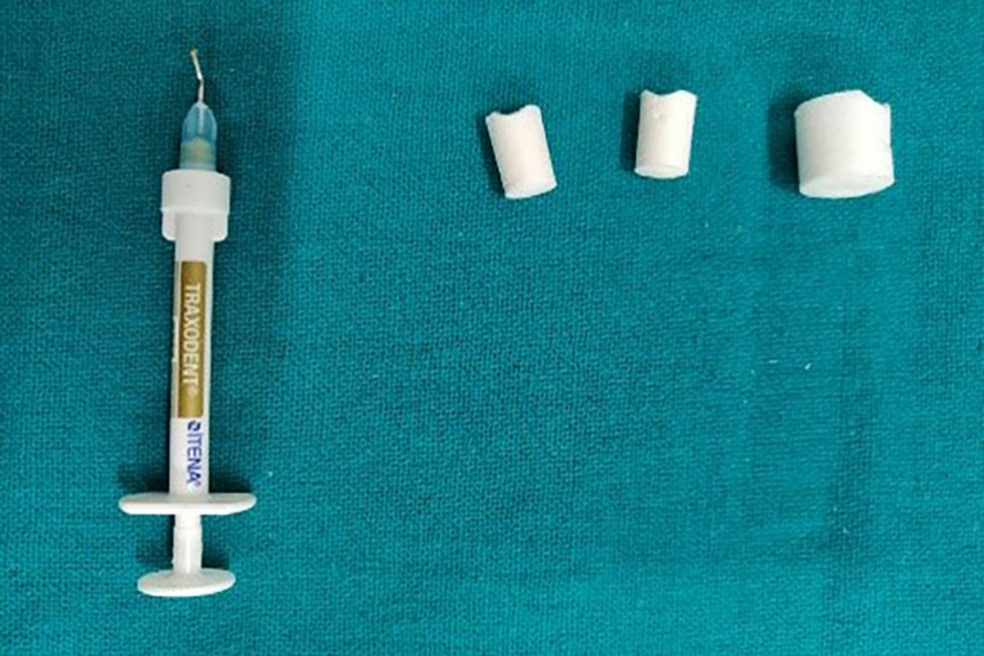
Retraction paste (Traxodent) with comprecaps

**Figure 9:**
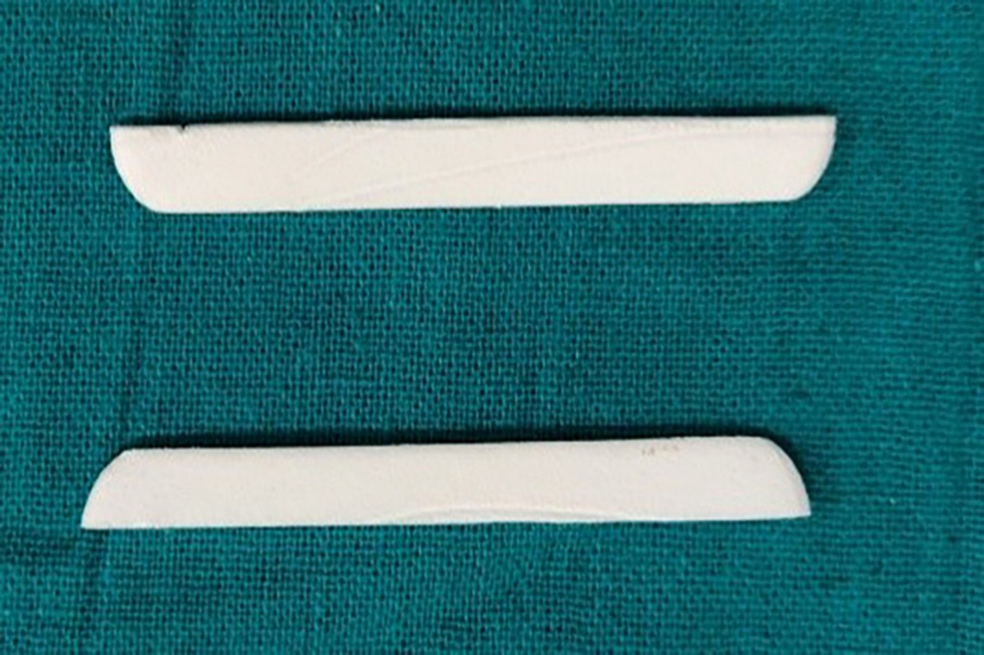
Polyvinyl acetate strips (Merocel)

Gingival retraction systems were divided into four groups. The first group received an impregnated gingival retraction cord (SURE-Cord), the second, a retraction capsule (3M ESPE), the third gingival retraction paste (Traxodent), and the fourth a polyvinyl acetate strip (Merocel) (Figures 6-9). To reduce bias to the greatest extent feasible, the four retraction systems were randomly evaluated on the right and left central incisors of all 20 participants. The test groups were then switched within-subjects 14 days after the first session.

**Figure 10:**
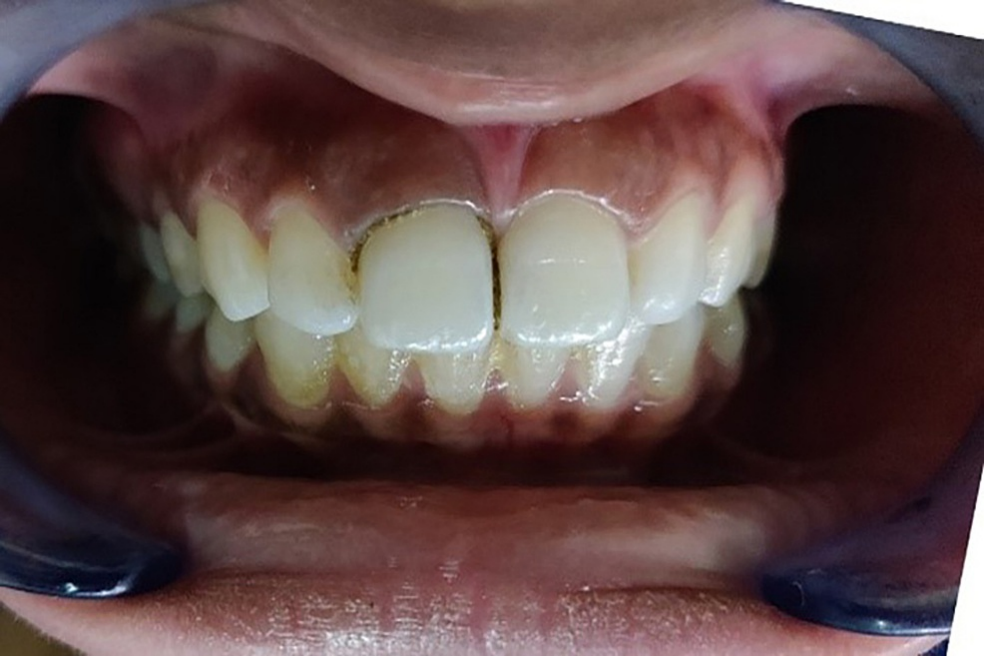
Gingival retraction with the retraction cord (SURE-Cord)

The maxillary anterior region was isolated with cotton rolls and air-dried using the three-way syringe in the case of Group 1, i.e., gingival retraction cord (SURE-Cord) (Figure 10). The Hu-Friedy Yardley gingival non-serrated cord packer (Hu-Friedy Mfg. Co., Chicago, IL) was used to place the required length of the retraction cord, beginning at the mesial interproximal area and progressing to the lingual surface and distolingual corner. The cord continued to be packed around the facial surface, overlying the cord in the mesial interproximal area. The instrument was slightly angled toward the tooth, allowing the cord to be placed straight into the sulcus. Care was taken not to injure the gingiva. The single cord technique was employed in this study. After two minutes, the cord was slowly removed, rinsed gently to eliminate any coagulum, and lightly blown on. Master impressions were made with the monophase addition silicone impression material as described above.

**Figure 11:**
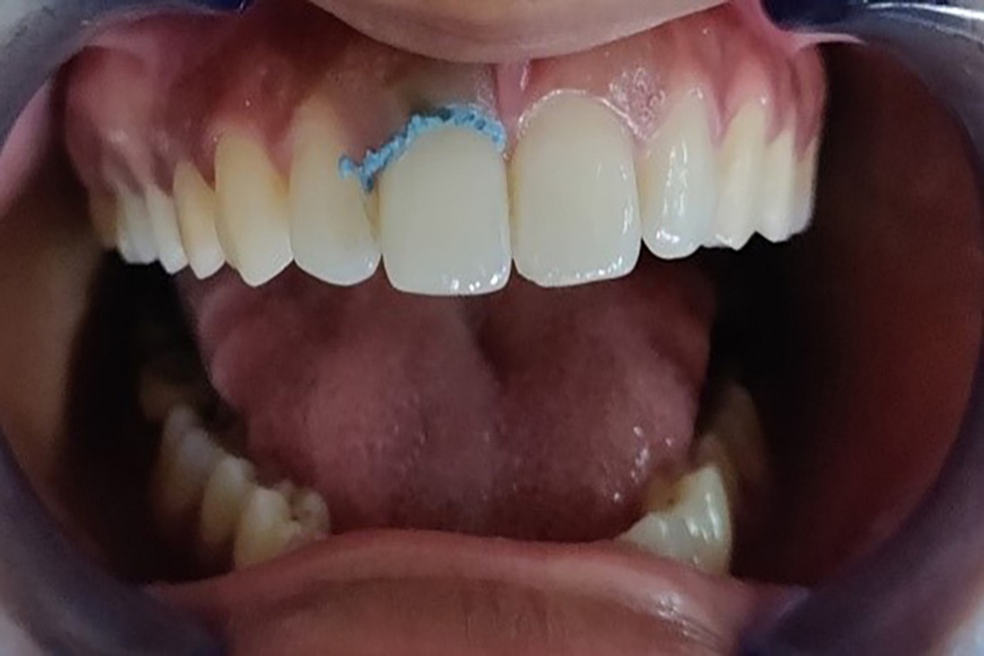
Gingival retraction with the retraction capsule (3M ESPE)

In the case of Group 2, i.e., retraction capsule (3M ESPE), teeth were isolated as described above, and the retraction paste was slowly injected into the sulcus from the capsule and left for two minutes as directed by the manufacturer (Figure 11). Blanching (from pink to white) of the marginal gingiva was observed to occur, and the impression of the displaced gingiva was made using the same monophase addition silicone impression material described above.

**Figure 12:**
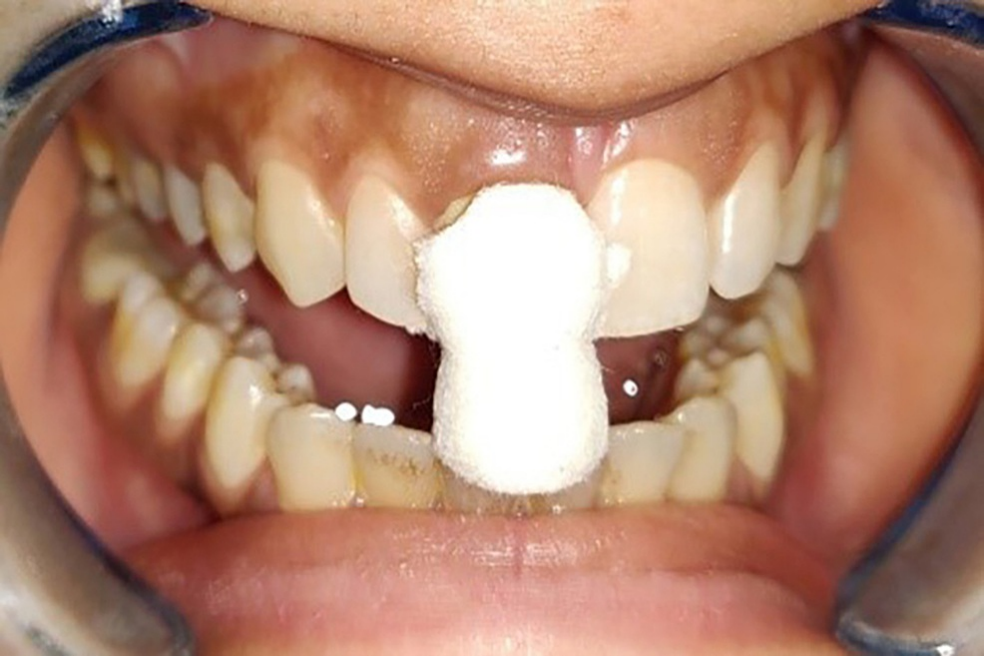
Gingival retraction with the retraction paste (Traxodent)

In the case of Group 3, i.e., gingival retraction paste (Traxodent, Itena), the retraction paste was gradually injected into the sulcus using a syringe (Figure 12). The correct size of the comprecap was chosen and customized, and it was used to hold the paste in place during the retraction procedure, with the patient directed to bite over it for two minutes per the manufacturer's instructions. Then an impression of the displaced gingiva was made with the monophase addition silicone impression material as described above.

**Figure 13:**
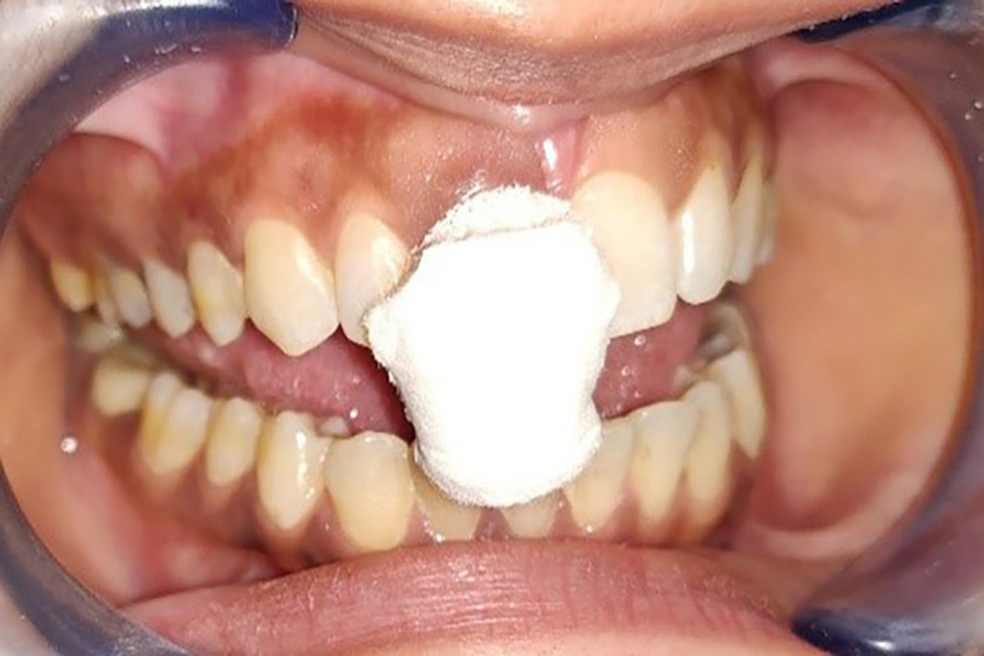
Gingival retraction with the polyvinyl acetate strip (Merocel)

**Figure 14:**
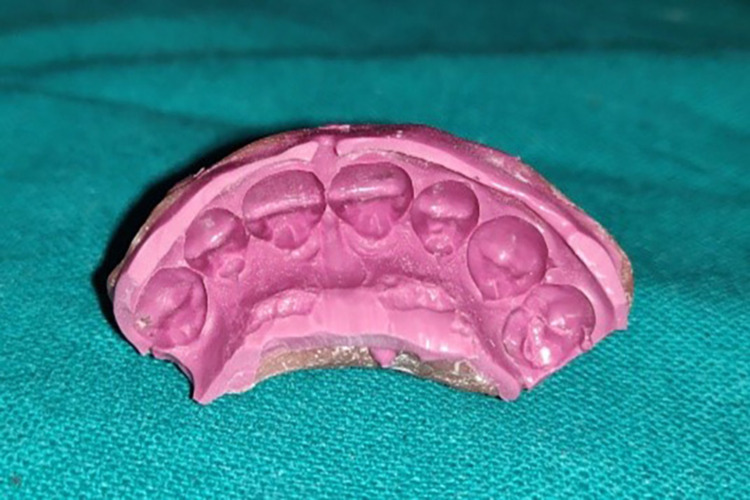
Post-gingival displacement impression

In the case of Group 4, i.e., polyvinyl acetate strip (Merocel), the Hu-Friedy Yardley non-serrated cord packer was used to place the polyvinyl acetate strip followed by the selected comprecap (Figure 13). The patient was then instructed to bite on it for two minutes. Before impression making, the Merocel strip and comprecap were discarded. Then an impression of the displaced gingiva was made with the monophase addition silicone impression material as described above (Figure 14). Patient comfort with these four retraction systems was evaluated subjectively by asking all subjects about their level of comfort with each of the retraction systems.

Step IV: Preparation of the casts for observations

**Figure 15:**
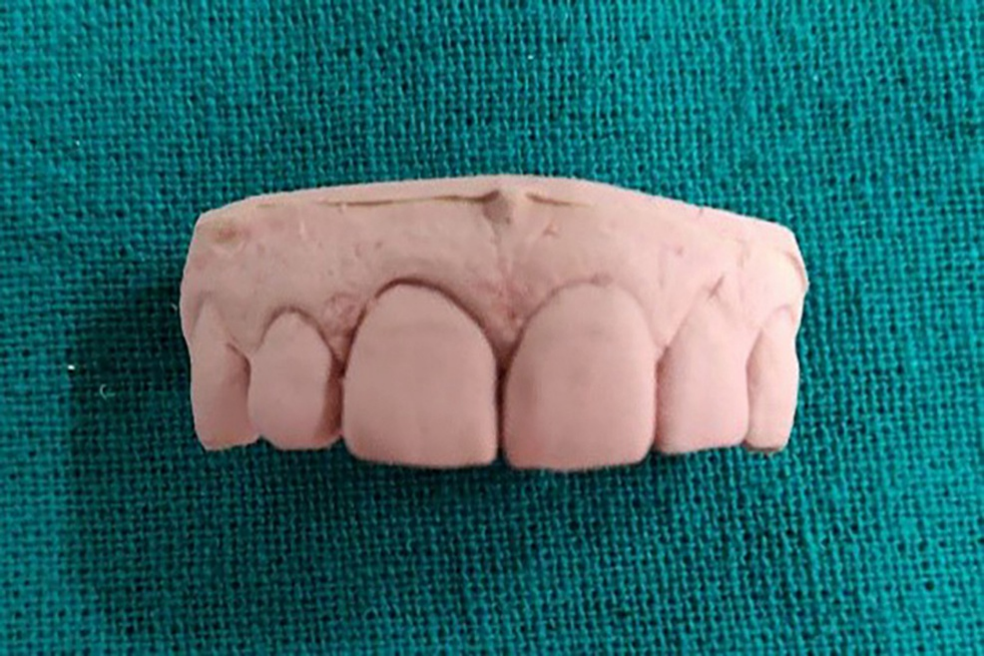
Post gingival displacement cast

**Figure 16:**
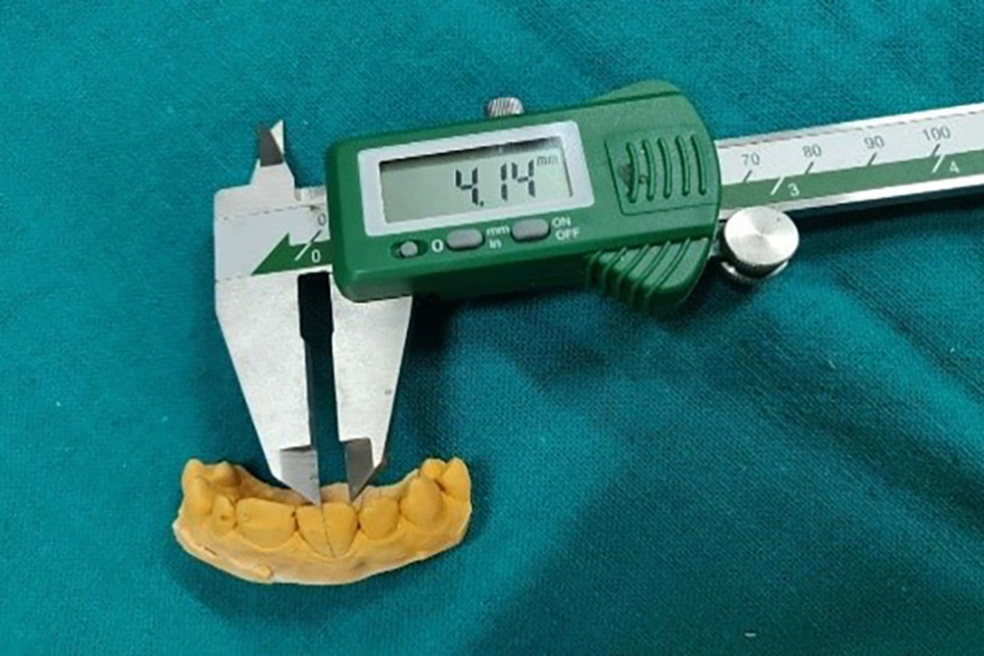
Measurement with Vernier's caliper

**Figure 17:**
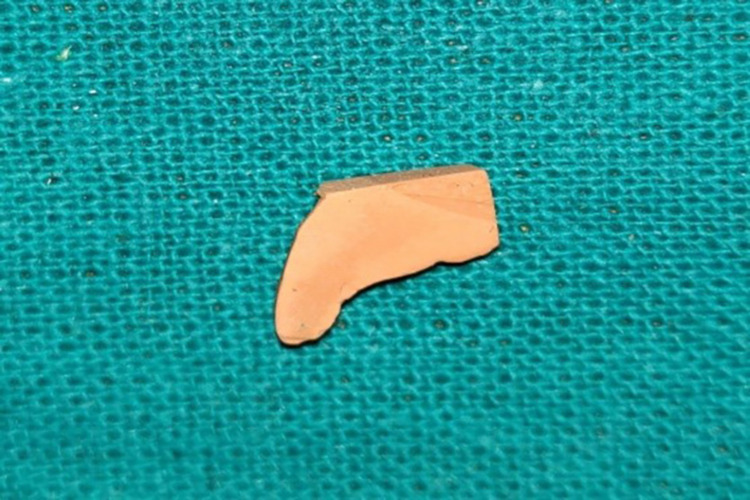
Section of the pre-gingival displacement cast

**Figure 18:**
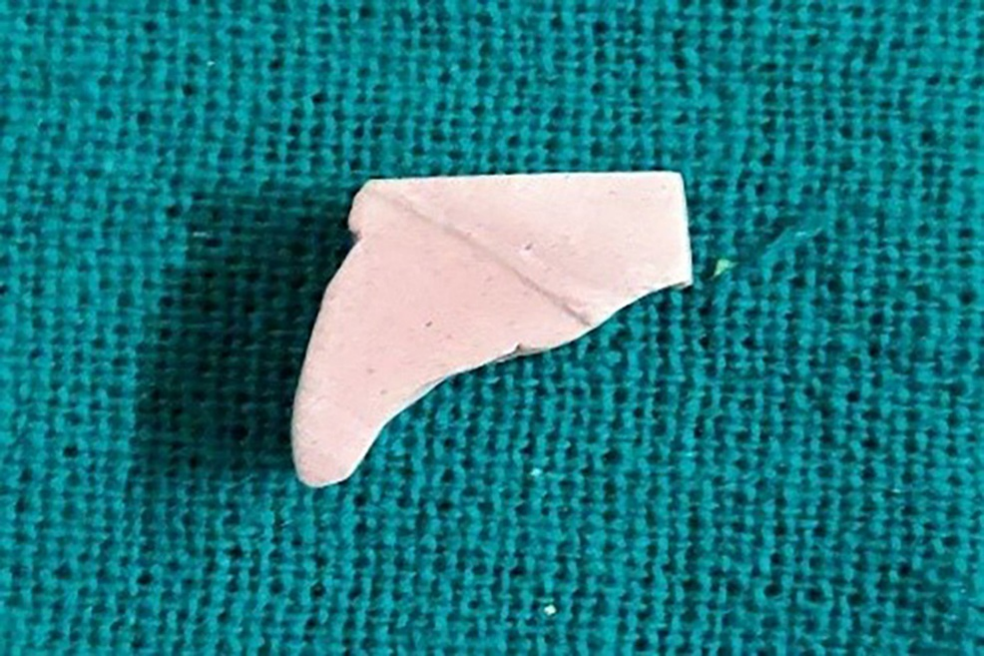
Section of post-gingival displacement cast

Pre-displacement and post-displacement impressions were poured with type IV dental stone (Figures 5, 15). After the final set, the casts were retrieved and trimmed to obtain a flat base. The midline of the maxillary central incisors on the buccal and palatal surfaces of the casts was marked with a digital caliper at the cervical and coronal levels (Figure 16). They were sectioned using a die-cutting machine in an apico-coronal direction, utilizing the midline as a reference point (Figures 17-18). As a result, each tooth had two halves, mesial and distal, that were used for measurement. The sectioned halves were examined using an optical microscope at a magnification of 5x using image analysis software.

Step V: Gingival tissue displacement measurement

**Figure 19:**
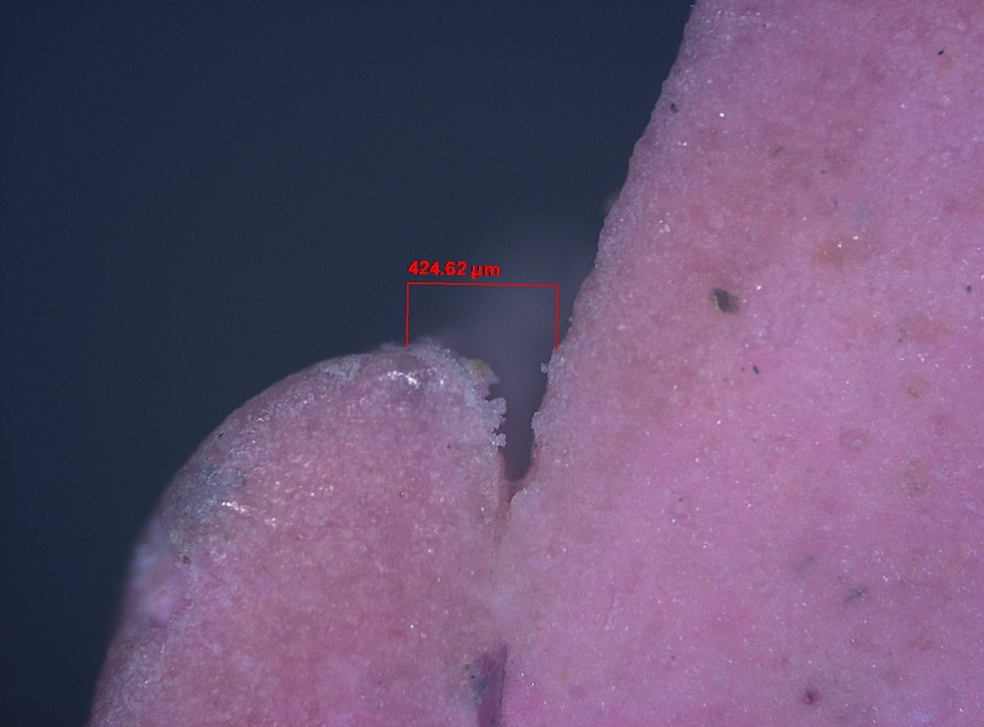
Digital image of gingival retraction by the retraction capsule (3M ESPE)

**Figure 20:**
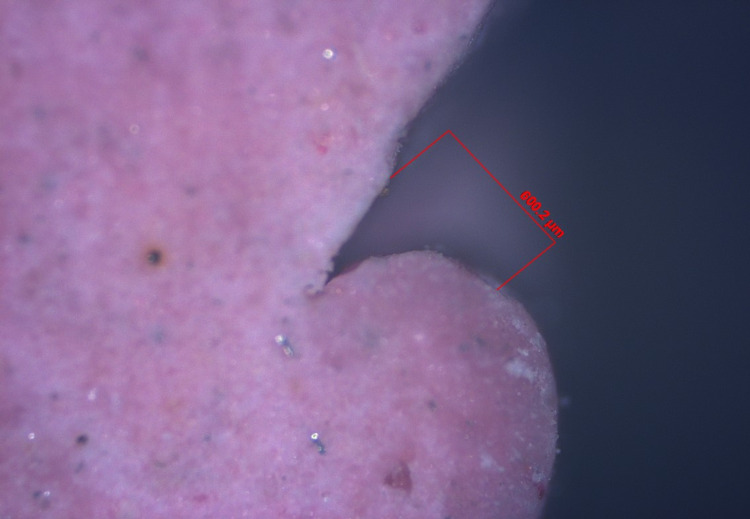
Digital image of gingival retraction by the retraction cord (SURE-Cord)

**Figure 21:**
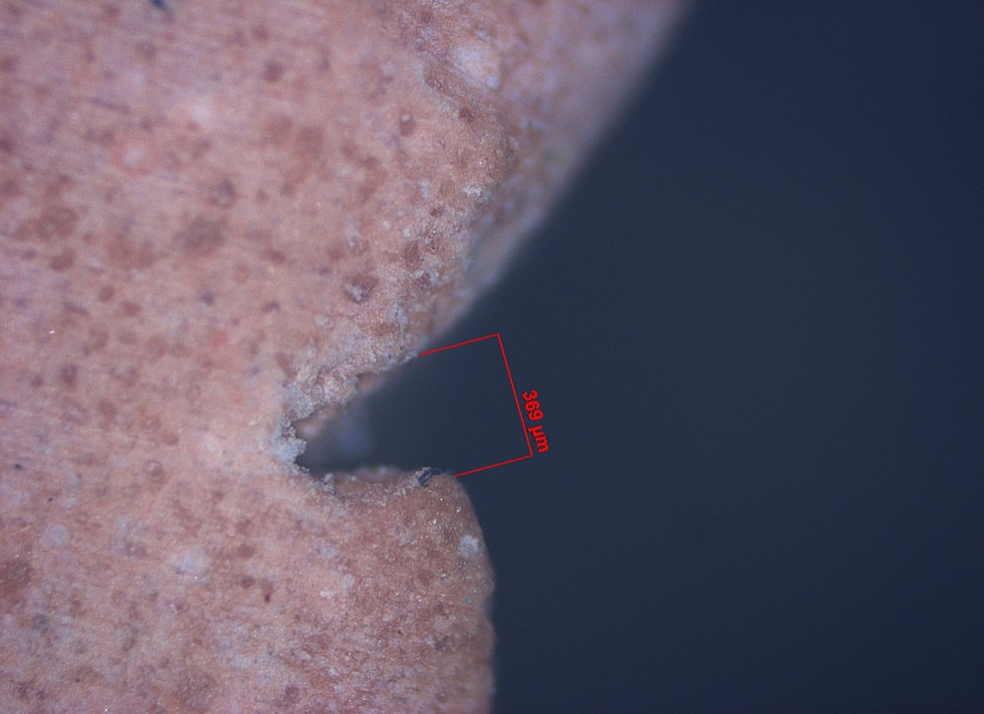
Digital image of gingival retraction by the retraction paste (Traxodent)

**Figure 22:**
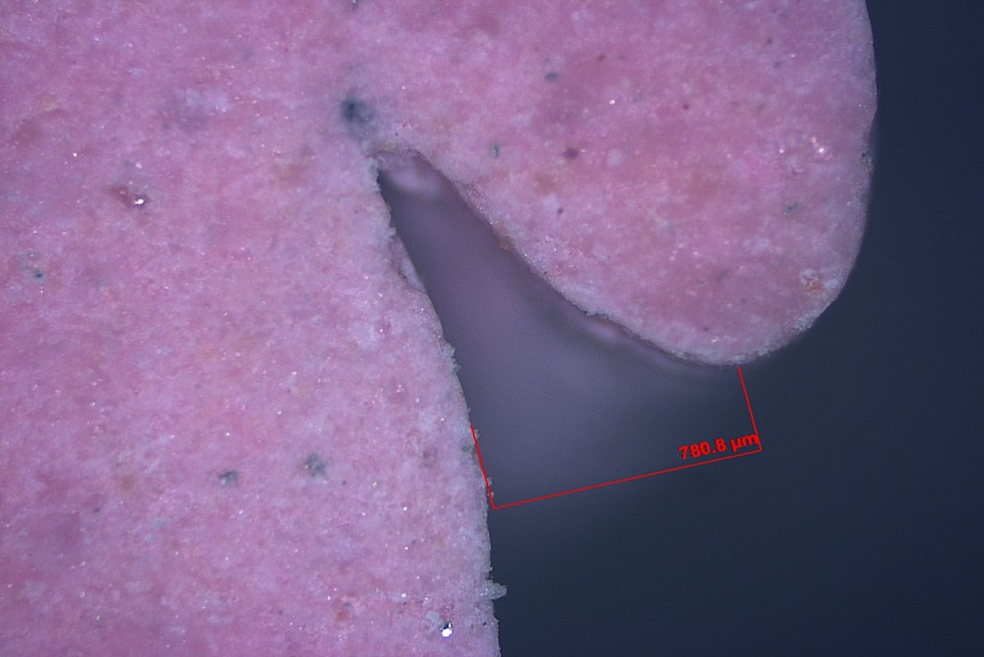
Digital image of gingival retraction by the polyvinyl acetate strip (Merocel)

The pre- and post-displacement sulcus width was measured for all specimens (Figures 19-22). The measurement was made from the crest of the gingival margin to the corresponding mid-buccal surface of the tooth. As a result, data on the width of the sulcus at the gingival margin's crest was obtained for both halves. The mean of these two values was used to calculate the reading at the mid-buccal half. The amount of lateral displacement was calculated from the obtained displacement values and post-displacement values at the mid-buccal position. The data was statistically analyzed using one-way analysis of variance (ANOVA), followed by a pair-wise comparison using a post hoc test.

Statistical analysis

The data obtained was compiled on an MS Office Excel Sheet, version 2019 (Microsoft, Redmond, WA). IBM SPSS Statistics, version 26.0 (IBM Corp, Armonk, NY) was used to analyze the data. Descriptive statistics like means and standard deviations (SDs) for numerical data have been depicted. An intergroup comparison of differences (>2 groups) was done using one-way ANOVA followed by pair-wise comparison using a post hoc test. For all the statistical tests, P<0.05 was considered to be statistically significant, keeping α error at 5% and β error at 20%, thus giving power to the study of 80%.

## Results

The means and SDs of the pre-displacement value for the mesial and distal halves were 164.10 ± 13.94 μm and 165.30 ± 10.97 μm. For the retraction capsule (3M ESPE), they were 495.58 ± 47.80 μm and 509.98 ± 41.42 μm, for retraction cord (SURE-Cord) 667.54 ± 47.93 μm and 672.624 ± 46.02 μm, for retraction paste (Traxodent) 393.49 ± 26.56 μm and 397.19 ± 22.92 μm and for polyvinyl acetate strip (Merocel), 705.83 ± 57.62 μm and 706.83 ± 57.62 μm (Table 1). The means and SDs of the gingival displacement value for retraction capsule (3M ESPE), retraction cord (SURE-Cord), retraction paste (Traxodent), and polyvinyl acetate strips (Merocel) were 333.57 μm ± 39.72, 505.37 μm ± 40.87, 230.63 μm ± 30.20 and 541.65 μm ± 44.73, respectively (Table 2). According to the ANOVA test, a statistically significant difference was noted between the groups concerning the mean lateral gingival retraction (P = 0.001) (Table 3).

**Table 1:** Average of pre- and post-displacement values in all four groups SD, standard deviation; ANOVA, analysis of variance **Statistically highly significant

Gingival retraction system	Mean	SD	Std. error	Lower bound	Upper bound	Minimum	Maximum	F value	P-value of one-way ANOVA
Pre-displacement	164.70 µm	12.01 µm	2.68 µm	159.08 µm	170.32 µm	130.83 µm	179.60 µm		
3M capsule	498.28 µm	43.37 µm	9.69 µm	477.98 µm	518.58 µm	421.46 µm	570.33 µm		
Retraction cord	670.08 µm	46.30 µm	10.35 µm	648.41 µm	691.75 µm	590.10 µm	739.23 µm	664.99	.000**
Traxodent paste	395.34 µm	23.40 µm	5.23 µm	384.38 µm	406.30 µm	353.27 µm	432.26 µm		
Merocel strips	706.35 µm	50.76 µm	1.35 µm	682.59 µm	730.11 µm	612.93 µm	867.21 µm		

**Table 2:** Intergroup comparison of values of gingival displacement for the four groups SD, standard deviation; ANOVA, analysis of variance **Statistically highly significant

Gingival retraction system	Mean	SD	Std. error	Lower bound	Upper bound	Minimum	Maximum	F value	P-value of one-way ANOVA
3M capsule	333.57 µm	39.72 µm	8.88 µm	314.98 µm	352.17 µm	257.97 µm	397.78 µm		
Retraction cord	505.37 µm	40.87 µm	9.13 µm	486.24 µm	524.50 µm	427.08 µm	569.58 µm	277.958	.000**
Traxodent paste	230.63 µm	30.20 µm	6.75 µm	216.50 µm	244.77 µm	173.86 µm	297.86 µm		
Merocel strips	541.65 µm	44.73 µm	10.00 µm	520.71 µm	562.59 µm	463.70 µm	687.60 µm		

**Table 3:** Intergroup pairwise comparison of values of gingival displacement for all four groups *Statistically significant **Statistically highly significant Group 1 = impregnated gingival retraction cord (SURE-Cord), Group 2 = gingival retraction capsule (3M ESPE), Group 3 = gingival retraction paste (Traxodent), Group 4 = polyvinyl acetate strip (Merocel)

Dependent variable	Group	Group	Mean difference	Std. error	P-value	Lower bound	Upper bound
Gingival displacement	1	2	-1.7180075E2 µm	12.4120869 µm	.000**	-204.404779 µm	-139.196721 µm
	1	3	102.9392500 µm	12.4120869 µm	.000**	70.335221 µm	135.543279 µm
	1	4	-2.0807300E2 µm	12.4120869 µm	.000**	-240.677029 µm	-175.468971 µm
	2	3	274.7400000 µm	12.4120869 µm	.000**	242.135971 µm	307.344029 µm
	2	4	-36.2722500 µm	12.4120869 µm	.023*	-68.876279 µm	-3.668221 µm
	3	4	-3.1101225E2 µm	12.4120869 µm	.000**	-343.616279 µm	-278.408221 µm

## Discussion

The clinical success of fixed prosthodontic restorations made in a dental laboratory is dependent on the final impression's accuracy. The location of the finish lines, periodontal health, and moisture control during impression making have an impact on the quality of the impression. Modern impression materials used in restorative dentistry necessitate gingival tissue displacement to expose and record the intracrevicular subgingival and/or equigingival finish lines on the tooth surface [22].

There are numerous gingival retraction systems on the market. This study compared the amount of lateral gingival retraction by four gingival retraction systems, namely, impregnated retraction cord (SURE-Cord), gingival retraction capsule (3M ESPE), retraction paste (Traxodent), and polyvinyl acetate strips (Merocel), in 20 subjects with unprepared maxillary central incisors (either left or right). Unprepared teeth were chosen to evaluate gingival displacement in the absence of external influences such as mechanical trauma and the inflammatory response of the gingiva after tooth preparation. For accurate gingival tissue details, a single-step impression was made in the custom tray using the monophase addition silicone impression material [19].

A 14-day interval was kept between the use of the two retraction systems to allow the gingiva to return to a healthy state. The SURE-Cord retraction cord was held in the sulcus for two minutes per the manufacturer's recommendations as the cord requires time to induce adequate lateral displacement, and the medication needs time to create hemostasis and manage crevicular fluid [23]. The recommended time, according to several studies, ranges from 1 to 30 minutes. Baharav et al. concluded that there was no significant difference in the crevicular width at any time of tissue displacement for four, six, or eight minutes with impregnated cords [24]. The retraction cord (SURE-Cord) is a knitted material with a stable structure. As the knitted loops seek to open, they mechanically displace the gingival tissue, absorb crevicular fluids, and exert a gentle, continuous outward force. Nonimpregnated cords are less appropriate for hemostasis than those impregnated with aluminium sulphate and epinephrine [25]. Soaking the cord in an aluminium chloride solution before inserting it into the gingival sulcus provides hemostasis without impairing the cord's ability to absorb crevicular fluid. Aluminum chloride causes vasoconstriction, which leads to gingival shrinkage. This astringent works by precipitating proteins, constricting blood vessels, and extracting fluid from tissues [25]. As a result, in this study, an aluminium chloride hemostatic agent was used with the least amount of force that did not affect the polymerization and accuracy of impression materials, as displacement can cause haemorrhage and damage to the sulcular and junctional epithelium. Before making the impression with vinyl polysiloxane, all traces of medicaments were carefully removed.

This study employed three cordless systems: polyvinyl acetate strips (Merocel), retraction paste (Traxodent), and retraction capsule (3M ESPE). The polyvinyl acetate strip is a synthetic and porous material that is chemically derived from a biocompatible polymer (hydroxylated polyvinyl acetate) and has a net-like structure. Because the material is pliable, it can be easily shaped and inserted into the sulcus. With the absorption of intracrevicular fluids, this sponge-like material expands, exerting moderate pressure on surrounding gingival tissues and ensuring gingival displacement.

The second cordless material used in this study was the retraction paste (Traxodent). The soft paste contains about 15% aluminium chloride. It exerts light pressure on the sulcus while absorbing excess crevicular fluid and blood. Aluminum chloride acts as an astringent without irritating or discolouring the surrounding tissue.

The third cordless material used was a retraction capsule (3M ESPE) containing an astringent paste with 15% aluminium chloride and designed to be used with a composite capsule dispenser. The capsule has a long, slim nozzle with a soft edge that allows the high-viscosity astringent paste containing 15% aluminium chloride to be delivered directly into the gingival sulcus. The nozzle also has a white orientation ring that corresponds to the size and position of the periodontal probe and prevents the delivery nozzle from impinging on the gingival sulcus.

In this study, an optical microscope with a magnification of 5x and image analysis software was used to measure lateral gingival retraction from a sectioned cast of a post-gingival displacement impression. The gingival sulcus' width was measured at the mid-buccal region of the sulcular extensions. The image analysis measurements were in micrometre grading.

The lateral gingival retraction mean and SD values for the retraction capsule (3M ESPE), impregnated retraction cord (SURE-Cord), retraction paste (Traxodent), and polyvinyl acetate strip (Merocel) were 333.57 μm ± 39.72, 505.37 μm ± 40.87, 230.63 μm ± 30.20 and 541.65 μm ± 44.73, respectively. The minimum and maximum values for the retraction capsule (3M ESPE) were 257.97 μm and 397.78 μm, for the impregnated retraction cord (SURE-Cord) were 427.08 μm and 569.58 μm, for the retraction paste (Traxodent) were 173.86 μm and 297.86 μm, and for the polyvinyl acetate strip (Merocel) were 463.70 μm and 687.60 μm. The one-way ANOVA test revealed that the means of lateral retraction differed significantly (F = 277.95, P<0.001). The post hoc test was used for multiple comparisons.

The retraction cord's displacement can be attributed to it being a 'chemicomechanical method' of gingival displacement. The enlargement of the gingival sulcus and control of fluids seeping from the gingival sulcus walls are made easier by combining chemical action with pressure packing. As the statistical analysis revealed a significant difference in the gingival retraction capacity between the materials tested, the null hypothesis was rejected. Within the limits of our study, polyvinyl acetate strips (Merocel) provided maximum gingival displacement, while the retraction paste (Traxodent) provided the least, which is consistent with studies by Shivasakthy et al., Zala et al., and Thimmappa et al., who concluded that polyvinyl acetate strips provided greater gingival displacement than retraction cord [17,26,27]. When compared to the SURE-Cord retraction cord, the polyvinyl acetate strip (Merocel) system showed great potential for haemorrhage control, moderate clinical time for application, and ease of placement. The porous and sponge-like microstructure of polyvinyl acetate strips (Merocel) absorbs intracrevicular fluid, ensuring a dry environment that allows for the adequate flow of the impression material to record precise tissue details [16,17].

However, the use of the polyvinyl acetate strip (Merocel) material as a gingival retraction device has some limitations, including the requirement for temporary crowns during the impression-making process because it is difficult to secure the material in place during the placement and retraction process. When the material is dry, it is easier to place than when it is wet. In the future, studies can be conducted by switching the Merocel form from strip to cord, making placement much easier. Using a reasonable adaptation of the technique to the clinical demand, it is quickly possible to uncover the gingival finish lines of the prepared tooth and record their margins in an impression without causing an iatrogenic tissue injury. Further research can be done to analyze the effect of the polyvinyl acetate strip on the health of the gingival sulcus epithelium and compare it with other contemporary materials. Although retraction pastes provided less gingival retraction, they were more than the minimum sufficient requirement of 200 μm. It was consistent with the findings of Zala et al., Tiwari et al., Sachdev et al., and Indriyani et al. [26,28-30]. They were less damaging to gingival health, took less time, and were more comfortable for the patient than retraction cords and Merocel strips. As a result, they can be used as an alternative to retraction cords.

Within the limitation of the present study, future research with a larger sample size is required to evaluate the gingival displacement in different clinical conditions like prepared teeth with subgingival margin, inflammatory soft tissues, and varying gingival thicknesses.

## Conclusions

In conclusion, polyvinyl acetate strips (Merocel) provided the maximum lateral gingival retraction among the four materials studied in this study. Retraction capsules (3M ESPE) and retraction paste (Traxodent) were easier to apply when compared to retraction cords (SURE-Cord) and polyvinyl acetate strips. The retraction capsule and retraction paste provided more patient comfort than the retraction cord and polyvinyl acetate strips, with the retraction paste providing the most and retraction cord providing the least comfort.
